# Prediction of CO_2_ solubility in Ionic liquids for CO_2_ capture using deep learning models

**DOI:** 10.1038/s41598-024-65499-y

**Published:** 2024-06-26

**Authors:** Mazhar Ali, Tooba Sarwar, Nabisab Mujawar Mubarak, Rama Rao Karri, Lubna Ghalib, Aisha Bibi, Shaukat Ali Mazari

**Affiliations:** 1 Department of Chemical Engineering, Dawood University of Engineering & Technology, Karachi, Pakistan; 2 Petroleum and Chemical Engineering, Faculty of Engineering, Universiti Teknologi Brunei, Bandar Seri Begawan, BE1410 Brunei Darussalam; 3 Department of Chemistry, School of Chemical Engineering and Physical Sciences, Lovely Professional University, Phagwara, Punjab 144411 India; 4 INTI International University, 71800 Nilai, Negeri Sembilan Malaysia; 5 Materials Engineering Department, Mustansiriayah University, Baghdad, 14022 Iraq; 6 Department of Education, NUML, Islamabad, Pakistan

**Keywords:** Ionic liquids, CO_2_ capture, Deep learning, ANN, LSTM, Global sensitivity analysis, Environmental sciences, Energy science and technology, Engineering, Materials science, Mathematics and computing

## Abstract

Ionic liquids (ILs) are highly effective for capturing carbon dioxide (CO_2_). The prediction of CO_2_ solubility in ILs is crucial for optimizing CO_2_ capture processes. This study investigates the use of deep learning models for CO_2_ solubility prediction in ILs with a comprehensive dataset of 10,116 CO_2_ solubility data in 164 kinds of ILs under different temperature and pressure conditions. Deep neural network models, including Artificial Neural Network (ANN) and Long Short-Term Memory (LSTM), were developed to predict CO_2_ solubility in ILs. The ANN and LSTM models demonstrated robust test accuracy in predicting CO_2_ solubility, with coefficient of determination (R^2^) values of 0.986 and 0.985, respectively. Both model's computational efficiency and cost were investigated, and the ANN model achieved reliable accuracy with a significantly lower computational time (approximately 30 times faster) than the LSTM model. A global sensitivity analysis (GSA) was performed to assess the influence of process parameters and associated functional groups on CO_2_ solubility. The sensitivity analysis results provided insights into the relative importance of input attributes on output variables (CO_2_ solubility) in ILs. The findings highlight the significant potential of deep learning models for streamlining the screening process of ILs for CO_2_ capture applications.

## Introduction

Carbon dioxide (CO_2_) released into the atmosphere through industrial production has resulted in significant environmental issues, including global climate change^1^. To mitigate the emission and accumulation of CO_2_, the capture and separation of CO_2_ from natural and flue gas have emerged as effective approaches^2^. Various technologies have been developed for CO_2_ separation, including amine scrubbing^3^, pressure swing adsorption (PSA)^4^, temperature swing adsorption (TSA)^5^, and membrane separation technology^6^. Among these technologies, amine absorption is widely utilized in industries. The commonly employed amine solvents for CO_2_ absorption include monoethanolamine (MEA), methyldiethanolamine (MDEA), and diethanolamine (DEA)^1^. However, these absorbents have limitations, such as being prone to volatility and demanding high energy consumption during desorption^7^. Traditional CO_2_ capture methods, like amine scrubbing, are hindered by their high energy demands for regeneration and significant solvent loss. This combination not only increases operational costs but also contributes to a larger environmental footprint^8^.

In the past decade, ionic liquids (ILs) have become the most potential applicants for CO_2_ capture. The utilization of ILs in carbon capture represents a favourable alternative to conventional amine-based solvents, primarily due to two key advantages: their remarkably low vapour pressure and the ability to tailor their molecular structure to suit specific requirements^9^. These remarkable achievements of ILs are due to their unique molecular structures (anions, cations, and functional groups) and exceptional properties such as thermal stability, nonvolatility, and outstanding CO_2_ solubility^10–14^. The general properties of the majority of ILs are presented in Table 1^15^.

**Table 1:** General properties of ILs^15^.

Property	General characters
Salt ions	Large cations and anions
Freezing temperature	< 100 °C
Liquidous temperature	> 200 °C
Thermal stability	High
Viscosity	< 100 cP, workable
Dielectric constant	< 30
Polarity	Moderate
Specific conductivity	< 10 mS/cm, good
Vapor pressure	Negligible
Solvency	Strong
Catalytical character	Excellent (for organic reactions)

One major challenge in utilizing ILs for CO_2_ capture is their high viscosity because of the complex synthesis and purification processes required to create ILs. Compared to conventional solvents typically used for CO_2_ capture, ILs generally exhibit significantly higher viscosity^16^. As highlighted by Krupiczka et al.^17^, the viscosity of ILs can be altered by employing appropriate combinations of cations and anions. Notably, the anion has a greater viscosity influence than the cation. Increasing the alkyl chain length within the cation generally leads to a corresponding increase in IL viscosity^17^. In terms of anion effects on viscosity in imidazolium based ILs, the reported order is [bmim][NTf_2_] < [bmim][CF_3_SO_3_] < [bmim][BF_4_] < [bmim][PF_6_]. ILs are highly adaptable and can be customized for specific applications by varying the types and ratios of cations and anions. This versatility serves as the basis for designing^18^.

The development of accurate models to predict the solubility of CO_2_ in ILs is a critical aspect of the design of ILs for carbon capture using computer-aided molecular design (CAMD). Traditional thermodynamic models have been utilized to estimate gas solubilities, including CO_2_, in ILs. Some of these models include the Peng–Robinson–Stryjek–Vera (PRSV) equation of state^19^, group contribution-based Statistical Associating Fluid Theory (SAFT)^20^, cubic equations of state combined with the UNIFAC (UNIQUAC Functional-group Activity Coefficients) method^21^, and COSMO-RS (Conductor-like Screening Model for Real Solvents)^22^. These models are developed on robust thermodynamic principles and can accurately assess the effects of temperature and pressure. However, their ability to deliver precise quantitative solubility predictions may sometimes be inadequate.

In addition to rigorous thermodynamic modelling, the quantitative structure–property relationship (QSPR) method provides another practical approach for predicting solubility. This method establishes a quantitative correlation between the property of interest and specific structural descriptors of the molecules. Group contribution (GC) methods, which utilize the occurrences of functional groups in the molecule as molecular descriptors, are commonly employed in CAMD. Linear GC models are suitable for specific properties, while nonlinear GC models are required for accurately predicting other properties. Recently, there has been significant advancement and broad adoption of machine learning (ML) models for developing complex nonlinear QSPR or GC models. These models have demonstrated their effectiveness in estimating various properties, including CO_2_ solubility^23^, H_2_S solubility^24^, and surface tension^25^. ML models have emerged as a powerful tool for CO_2_ capture research. Their ability to learn from data allows them to rapidly predict complex material properties, like CO_2_ solubility in ILs^23^. This reduces the time and cost associated with traditional methods and provides valuable insights into the key factors governing CO_2_ capture efficiency^26^.

Neural network-based machine learning models have gained significant popularity in predictive analytics, particularly for estimating CO_2_ solubility. Eslamimanesh et al.^27^ designed an artificial neural network (ANN) model to predict the solubility of CO_2_ in 24 commonly used ILs for a dataset consisting of 1128 data points. Venkatraman and Alsberg^28^ applied various machine learning algorithms such as Partial-Least-Squares Regression (PLSR), Conditional Inference Trees (CTREE), and Random Forest (RF) to a dataset comprising 10,848 solubility measurements with 185 ILs. Soleimani et al.^29^ applied a decision tree-based stochastic gradient boosting (SGB) algorithm to predict H_2_S solubility in ILs using 465 experimental data points. Song et al.^30^ have developed ANN-GC and support vector machine (SVM-GC) models for CO_2_ solubility prediction in ILs with data containing 10,116 data points (with 124 different ILs). Deng et al.^31^ used three deep-learning models to predict CO_2_ solubility in ILs. They used a Convolutional Neural Network (CNN), Deep Neural Network (DNN), and a Recurrent Neural Network (RNN) with a relatively small dataset of 218 data points for 13 types of ILs. Recently, Tian et al.^32^ utilized ionic fragment contribution (IFC) with ANN and SVM models to predict CO_2_ solubility data with 13,055 instances in 164 kinds of ILs. Liu et al.^33^ estimated the CO_2_ solubility of 1517 data in 20 different ILs using Particle Swarm Optimization (PSO), Grey Wolf Optimization (GWO), and Sparrow Search Algorithm (SSA) based on SVM ML models. These models achieved higher prediction accuracy depending on the algorithm architecture and the associated dataset. Smaller datasets, which are less complex to train, generally achieve higher accuracy than larger datasets. DNNs excel at handling large datasets due to their ability to learn complex patterns with a higher number of neurons. However, for optimal performance with extensive data, optimization and regularization techniques become crucial to prevent overfitting.

This study aims to develop deep neural network-based models for the larger dataset to predict CO_2_ solubility in ILs. An ANN model and a long short-term memory (LSTM)-RNNs architecture are employed to address CO_2_ solubility prediction on this extensive dataset, which contains 10,116 CO_2_ solubility measurements from the work of Song et al.^30^. In their study, a simple ANN model with one hidden layer (8 neurons) was implemented on this data. However, their work lacks information about validation, hyperparameter tuning, and regularization techniques for such a large dataset of CO_2_ solubilities.

This work builds upon the previous study^30^ by proposing a DNNs-based ANN model with three hidden layers, each containing 64 neurons. Model validation and hyperparameter tuning were performed to assess the model's performance. The effectiveness of both the ANN and LSTM models was assessed based on computational costs and memory usage during model training. Furthermore, global sensitivity analysis tools, such as Sobol and Morris methods, were used to investigate the impact of various variables (including functional groups) on CO_2_ solubility in different ILs. ILs are promising for capturing CO_2_ emissions from power plants and industrial processes. By accurately predicting CO_2_ solubility, researchers design ILs with optimal CO_2_ capture capacity, leading to more efficient CCS technologies.

## Methods

### Dataset/experimental data

This study utilizes CO_2_ solubility data originally collected by Venkatraman and Alsberg^28^ and meticulously preprocessed and compiled by Song et al.^30^ for machine learning model training. The quality of the preprocessed data rendered further modifications unnecessary for our current analysis. This dataset includes 10,116 data points with 53 features that predict CO_2_ solubility in ILs. It covers 124 ILs across a temperature range of 243.2 K to 453.15 K and a pressure range of 0.00798 bar to 499 bar. The cations include imidazolium, pyridinium, piperidinium, pyrrolidinium, phosphonium, sulfonium, and ammonium. The anions include tetrafluoroborate [BF_4_], dicyanamide [DCA], hexafluorophosphate [PF_6_], chloride [Cl], nitrate [NO_3_], tricyanomethanide [C(CN)_3_], thiocyanate [SCN], bis(trifluoromethylsulflonyl)amide [Tf_2_N], hydrogen sulfate [HSO_4_], and methylsulfate [MeSO_4_] etc.

This study aims to develop deep-learning models for predicting CO_2_ solubility in ILs. Previous research by Song et al.^30^ on this dataset has not adequately addressed the optimization and regularization of neural network modelling. Our study fills this gap by focusing on several critical aspects, including model validation, hyperparameter tuning, computational efficiency, and the impact of neuron configurations on model performance. The modelling will use temperature, pressure (considered the most important features in CO_2_ capture due to their direct impact on IL performance), and other relevant factors (referred to as input parameters) to predict CO_2_ solubility (the output). The dataset of 10,116 data points is divided into training (80%) and testing (20%) sets to develop the deep learning models. This means the training set contains 8093 data points (80%), and the testing set contains 2023 (20%). During the model's training, 10% of the data was set aside for validation to ensure optimal performance. This dedicated validation set enabled monitoring of the model's validation loss curves throughout the training process. By analyzing these curves, potential overfitting could be identified, allowing for necessary adjustments to the model's architecture or training parameters.

### Model development

This section delves into the development of two deep learning models—an ANN and an LSTM-RNN network—for predicting CO_2_ solubility in ILs.

#### Artificial neural network (ANN)

**Figure 1:**
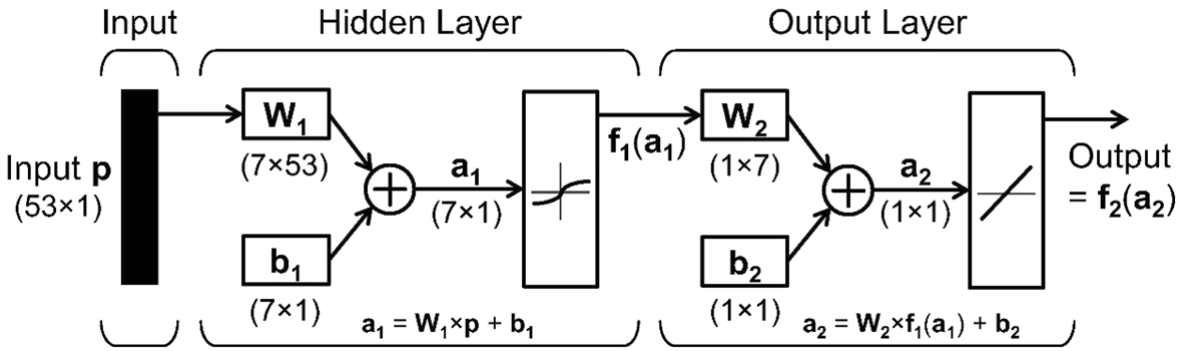
Schematic structure of ANN model^30^.

ANN is a biologically inspired network of artificial neurons modelled to perform various tasks^34^. These tasks include regression^35^, classification^36^, verification, and recognition. ANN model can recognize nonlinear complex relationships and can be used to predict CO_2_ solubility^37^. The literature indicates that various studies have used an ANN model to predict CO_2_ solubility in ILs^37–40^. An ANN model consists of different layers and certain numbers of neurons in each layer. As a feed-forward neural network, ANN consists of three layers—input, hidden, and output. The topology is shown in Fig. 1.

The input layer receives 53 features consisting of temperature, pressure, and functional groups, which gives an input vector *p* with a size of (53 $\times$ 1). The function of the hidden layer is to transfer this input information to the output layers where solubility can be predicted. The output from a hidden layer ${f}_{1}\left({a}_{1}\right)$ is demonstrated in Eqs. (1) and (2) define the output of the output layer.

1 $${f}_{1}\left({a}_{1}\right)={f}_{1}\left({W}_{1} \times p \times {b}_{1}\right)$$

2 $${f}_{2}\left({a}_{2}\right)={f}_{2}\left({W}_{2}\times {f}_{1}\left({a}_{1}\right)+{b}_{2}\right)$$

The ANN architecture comprises one input layer, one output layer, and three hidden layers. Each hidden layer is equipped with 64 neurons to optimize the model accuracy (see Supplementary Fig. S1). A detailed discussion regarding the adjustment of neurons is presented in "ANN model". The activation functions are used for the hidden and output layers. The primary role of these transfer functions is to transform the summed weighted input from the node into the output value for the next layer. In other words, the basic principle of function is to decide whether the neuron’s input is necessary in predicting data. Different activation functions are used for the neural network, such as the Sigmoid function, Tanh function (hyperbolic tangent), rectified linear activation function (ReLU), SoftMax, etc. The present study used ReLU for both hidden and output layers. A ReLU is a type of linear function. It is computationally more efficient than sigmoid and tanh functions due to its certain numbers of neuron activation since it doesn’t activate all neurons at the same time^41^. The mathematical expression for the ReLU function is given below.

3 $$f\left(x\right)=max (0,x)$$

#### Long short-term memory (LSTM) model

**Figure 2:**
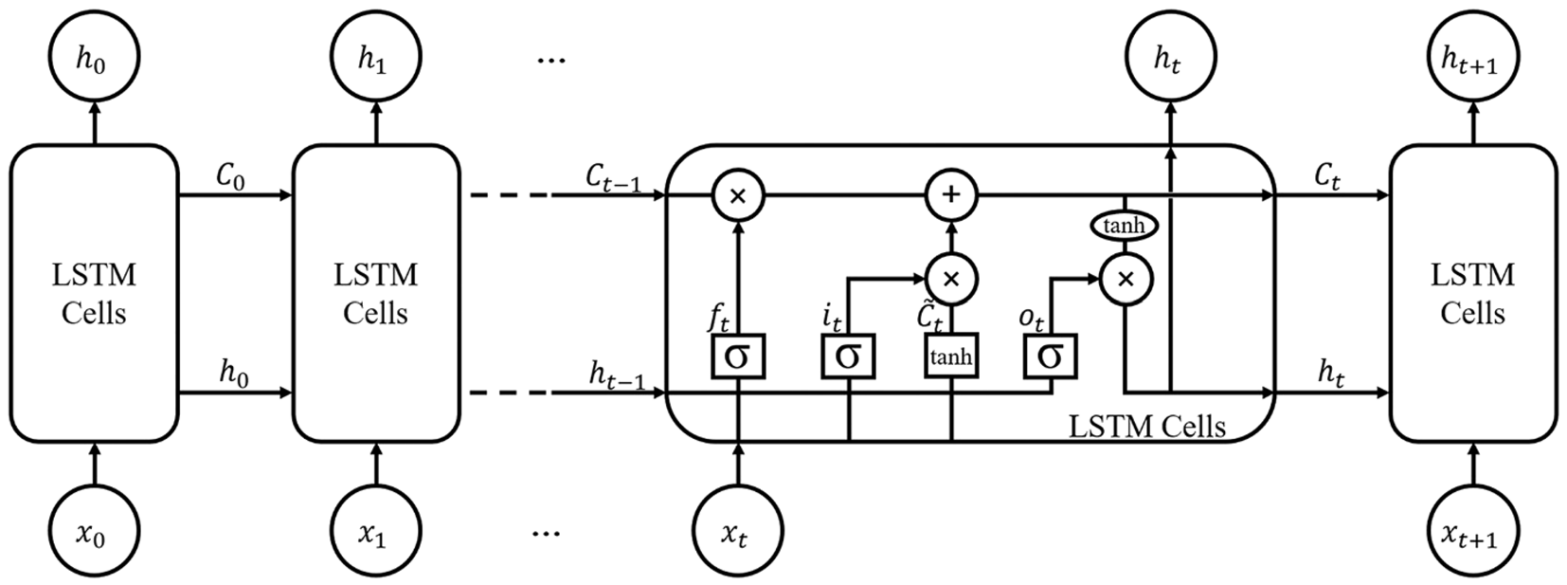
Basic LSTM layer structure^45^.

An LSTM is a special type of RNN architecture. RNN model performs poorly on long-term dependencies due to the vanishing gradient problem^42^. The LSTM is an extension model of RNN that uses memory structures to learn long-term information. These models can efficiently remove gradient problems^43,44^. The LSTM model gathers important information from input and saves this information for a long period, which is stored by a memory cell in the LSTM unit. A simple LSTM unit contains a cell, an input gate, a forget gate, and an output gate, as shown in Fig. 2. The cell remembers the values over arbitrary time intervals. The input gate decides which information should be added to the memory cell, while the forget gate decides whether to remove/save that information. Lastly, the output gate decides whether the existing information should have proceeded for analysis. Each LSTM cell contains six components in each timestep: a forget gate $"f"$ (a neural network with sigmoid function), a candidate layer $"C"$ (a neural network with tanh function), an input gate $"i"$ (a neural network with sigmoid), an output gate $"O"$ (a neural network with sigmoid), a hidden state $"h"$ (a vector) and a memory state $"\widetilde{C}"$ (a vector) as shown in Eqs. (4) to (9). The first parameter is the forget gate parameter $({f}_{t})$ that decides linear calculation based ${x}_{t}$ (current input) and ${h}_{t-1}$ (previous hidden state) values. The value of output for this gate is between 0 and 1, where 0 means the previous memory state is completely forgotten; else, if the value is 1, then it means the previous memory state is completely passed to the cell. The second parameter is the input gate, which contains two layers (a sigmoid layer and a tanh layer). The sigmoid layer decides regarding an update of values, and the tanh creates the addition of a new vector candidate $({\widetilde{C}}_{t})$ values to the LSTM memory. The output for these values is obtained with the help of Eqs. (5) and (6) and with the help of these, the cell state ${(C}_{t})$ is updated with the help of Eq. (7). Equation (8) helps to calculate the output parameter ${(o}_{t})$. The final output result at hidden state ${h}_{t}$ It can be obtained by using Eq. (9)

4 $${f}_{t}= \sigma \left({W}_{f}.\left[{h}_{t-1},{x}_{t}\right]+{b}_{f}\right)$$

5 $${i}_{t}=\sigma \left({W}_{i}.\left[{h}_{t-1},{x}_{t}\right]+{b}_{i}\right)$$

6 $${\widetilde{C}}_{t}=\text{tanh}\left({W}_{C}.\left[{h}_{t-1},{x}_{t}\right]+{b}_{C}\right)$$

7 $${C}_{t}={f}_{t}\times {C}_{t-1}+{i}_{t}\times {\widetilde{C}}_{t}$$

8 $${o}_{t}= \sigma \left({W}_{o}.\left[{h}_{t-1},{x}_{t}\right]+{b}_{o}\right)$$

9 $${h}_{t}={o}_{t}\times \text{tanh}\left({C}_{t}\right)$$

The LSTM architecture comprises an input layer, two hidden layers of 64 neurons, and an output layer (see Supplementary Fig. S1). While RNN architectures exist, this study employs LSTM networks due to their well-established capability to handle sequential data with long-term dependencies. In CO_2_ solubility prediction, the relationship between past and present data points can be crucial, especially when considering factors like temperature history or pressure fluctuations. Unlike simpler RNNs that struggle with vanishing gradients, LSTMs incorporate memory cells and gates that effectively capture and utilize these long-term dependencies, leading to potentially more accurate CO_2_ solubility predictions.

#### Sobol sensitivity analysis

Sobol sensitivity analysis, introduced by Sobol^46^ is a variance-based method that offers a global perspective. It aims to determine the contribution of each parameter and the interactions among parameters to the variance observed in the model output. Generally, the allocation of the overall output variance to individual model parameters and their interactions are written as

10 $$D\left(f\right)=\sum_{i}{D}_{i}+\sum_{i<j}{D}_{ij}+\sum_{i<j<k}{D}_{ijk}+{D}_{12\dots p},$$

where $D(f )$ represents the total variance of the output metric $f;$ ${D}_{i}$ is the first-order variance contribution of the ${i}^{th}$ parameter, ${D}_{ij}$ is the second-order contribution of the interaction between parameters $i$ and $j$; and ${D}_{12\dots p}$ contains all interactions higher than third-order, up to $p$ total parameters.

The first-order and total-order sensitivity indices are defined as follows.

First-order index:

11 $${S}_{i}=\frac{{D}_{i}}{D}$$

Total order index:

12 $${S}_{{T}_{i}}=1-\frac{{D}_{\sim i}}{D}$$

The first-order index captures the relative contribution of the parameter $i$ to the total output variance, excluding any effects or interactions with other parameters. The total order index equals one minus the fraction of the total variance assigned to ${D}_{i}$, which includes all parameters except $i$. By excluding parameter $i$ from the analysis, the total order index attributes the decrease in variance to that specific parameter^47^. The difference between a parameter’s first and total order indices corresponds to the impact of its interactions with other parameters.

This study analyzes the total order indices to ascertain the relative importance of model parameters regarding sensitivity. Total order indices, obtained through Sobol sensitivity analysis, capture the combined impact of each input parameter on the model output, accounting for both individual effects and interactions with other parameters. This analysis is crucial for identifying the parameters that significantly influence the variation in predicted CO_2_ solubility. Alternative sensitivity analysis methods might not provide the same level of detail. For instance, Morris sensitivity analysis, while efficient for initial screening, might not offer the in-depth information about individual and interactive effects that Sobol sensitivity analysis provides through total order indices. To ensure the robustness of our findings, we also employed the Morris method, allowing us to compare and select the most effective approach.

#### Morris sensitivity analysis

The method of Morris^48^ calculates global sensitivity measures by utilizing a set of local derivatives, also known as elementary effects. These effects are sampled on a grid that covers the parameter space. The method is based on a one-at-a-time (OAT) approach, where each parameter, denoted as ${x}_{i}$, is perturbed along a grid with a step size of ${\Delta }_{i}$. This perturbation allows for the creation of a trajectory through the parameter space, enabling sensitivity analysis across different parameter values. In a model consisting of $p$ parameters, a single trajectory comprises a sequence of $p$ perturbations. Each trajectory provides an estimate of the elementary effect for each parameter, which is determined by the ratio of the change in the model output to the change in the respective parameter. Equation (13) demonstrates the computation of a single elementary effect for the ${i}^{th}$ parameter.

13 $$E{E}_{i}=\frac{f\left({x}_{1},\dots ,{x}_{i}+{\Delta }_{i},\dots ,{x}_{p}\right)-f\left(x\right)}{{\Delta }_{i}}$$

where $f (x)$ represents the prior point in the trajectory. In alternative formulations, both the numerator and denominator in the calculation are normalized by the values of the function and parameter ${x}_{i}$, respectively, at a reference or prior point $x$^49^. This normalization ensures that the elementary effect is expressed relative to the function and parameter values at the reference point. Employing the single trajectory presented in Eq. (8) makes it possible to compute the elementary effects for each parameter with just *p* + *1* model evaluations. Nevertheless, since this one-at-a-time (OAT) method relies solely on a single trajectory, its results heavily rely on the initial point $x$ location within the parameter space and do not account for interactions between parameters. To address this limitation, the Morris^48^ extends the OAT method by conducting it across *N* trajectories throughout the parameter space.

The Morris method relies on the concept of elementary effects. These effects represent the change in the model output (predicted CO_2_ solubility) caused by small perturbations to a single input parameter across different points in the parameter space. The Morris method utilizes a grid-based approach to compute elementary effects. It repeatedly samples the parameter space, slightly increasing or decreasing the value of a single parameter at each sample point while keeping all other parameters fixed. The difference between the model outputs obtained with the original and perturbed parameter value is the corresponding elementary effect^48^. The mean effect (*μ*) defines a parameter's average of elementary effects and indicates its overall influence on the model output. A positive value suggests the parameter generally increases CO_2_ solubility, while a negative value indicates the opposite.

### Statistical indexes as an error function

In this section, the reliability and accuracy of the predicted models were evaluated through statistical analysis. Five key statistical indexes were determined: coefficient of determination (R^2^), root mean square error (RMSE), mean squared error (MSE), mean absolute error (MAE), and average absolute relative deviation (AARD). These indexes provide a comprehensive assessment of the model's performance and ability to predict CO_2_ solubility accurately.

14 $${R}^{2}= 1-\frac{{\sum }_{i-1}^{N}{\left({X}_{i}^{exp}-{X}_{i}^{predicted}\right)}^{2}}{{\sum }_{i-1}^{N}{\left({X}_{i}^{exp}-\overline{{X }_{i}^{exp}}\right)}^{2}}$$

15 $$RMSE= \sqrt{\left(\frac{1}{N}\sum_{i=1}^{N}{\left({X}_{i}^{exp}-{X}_{i}^{predicted}\right)}^{2} \right)}$$

16 $$MSE= \frac{1}{N}\sum_{i=1}^{N}{\left({X}_{i}^{exp}-{X}_{i}^{predicted}\right)}^{2}$$

17 $$MAE= \frac{1}{N}\sum_{i=1}^{N}{X}_{i}^{exp}-{X}_{i}^{predicted}$$

18 $$AARD=\frac{100}{N}\sum_{i=1}^{N}\left|\frac{{X}_{i}^{exp}-{X}_{i}^{predicted}}{{X}_{i}^{exp}}\right|$$

## Results and discussion

### ANN model

The performance efficiency in predicting CO_2_ solubility was different for different models while considering the same parameters and optimization methods for prediction. A better choice of optimizer was needed to modify the attributes of the neural network models. Ruder^50^ in their comprehensive review of modern optimization algorithms and recommended ‘Adam’ as the superior choice among various optimizer techniques; hence, Adam optimizers, coupled with the ReLU activation function, were employed for each model to achieve optimized efficiency.

In neural network modelling, the learning rate is a crucial hyperparameter that influences how the model updates its weights during training. A well-chosen learning rate ensures the model learns effectively, at neither too slow a pace (which can lead to underestimation) nor too quickly (which can cause overestimation). A learning rate of 0.001 was selected within the tested range because further decrease resulted in a significant decline in model performance.

A critical step in neural network design is determining the ideal number of neurons in hidden layers. Having too few neurons can lead to underfitting, where the model fails to capture crucial patterns in the data. Conversely, too many neurons can cause overfitting, where the model memorizes noise instead of learning the underlying relationships. This study began with an architecture containing 8 neurons per hidden layer. We then systematically increased this number to 64 neurons per layer, searching for the optimal balance between underfitting and overfitting (see Fig. 14). The ANN model incorporates three hidden layers, each containing 64 neurons. A visual representation of this architecture, generated using the NETRON tool^51^, is provided in Supplementary Fig. S1 of the Supplementary Material.

Model validation was performed to verify the accuracy and fit of the model. The training and validation loss functions served as metrics for evaluating the efficiency of the ANN model. The training loss measured how effectively the model fits the training data, while the validation loss indicated its ability to fit new data. The training loss function served as a metric to gauge how effectively the model learned the patterns within the training data, and the validation loss function assessed the ability to generalize these patterns and fit test data. Ideally, the training loss should decrease as the model learns.

**Figure 3:**
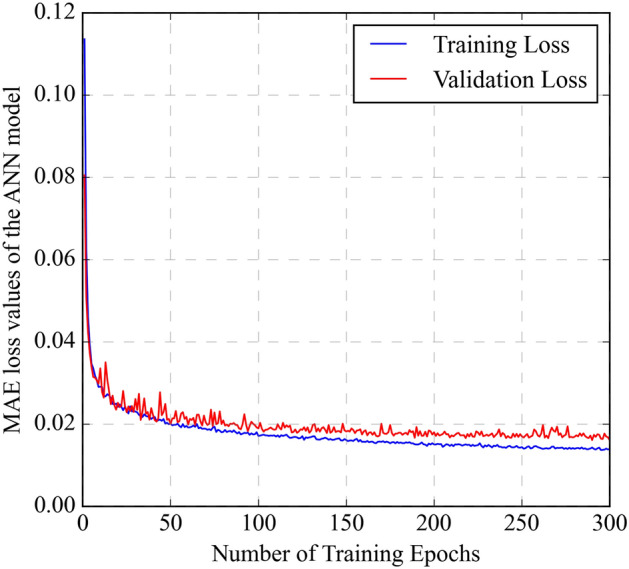
Mean absolute error (MAE) loss curves for the LSTM model showing training and validation performance over epochs.

In contrast, the validation loss should remain stable or slightly increase, indicating that the model avoids overfitting the training data. Figure 3 illustrates the training and validation loss curves using the MAE metric. The MAE loss curves depict a significant decrease in training loss (blue line), indicating successful learning of the ANN model for the training data. The validation loss (red line) remains stable, suggesting that the model avoids overfitting and generalizes well to new data.

**Figure 4:**
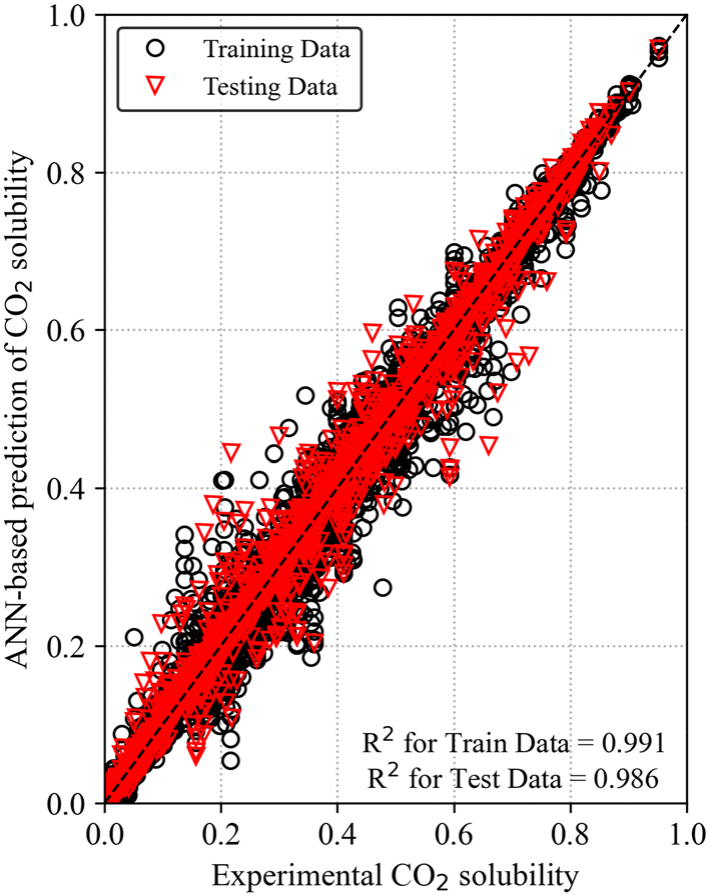
Comparison of actual and ANN-predicted CO_2_ solubility.

This study aims to optimize the performance of the ANN model by utilizing the different activation functions and a higher number of neurons compared to the previous study^30^ for this dataset. The ANN model exhibited improved performance with an increased number of neurons. The higher number of neurons enables the network to understand complex decision boundaries better and express a broader spectrum of functions, ultimately leading to an improved model capacity^52,53^. Table 2 summarizes the performance of the ANN model on the training (8,093 data points) and testing datasets (2,023 data points) using R^2^, MAE, RMSE, MSE, and AARD metrics. The R^2^ of 0.986 and MAE of 0.0171 indicate a good fit between the predictions and the experimental values. The ANN model showed a decrease in MSE values as the number of neurons in the hidden layers increased, indicating that a more complex architecture enhanced its learning capability. Figure 4 visualizes the comparison between the actual and predicted CO_2_ solubility values for both the training and testing sets. It is evident from Fig. 4 that both the training and testing datasets exhibit a strong relationship with the diagonal line, indicating a good fit with the experimental CO_2_ solubility data. However, a few outliers are observed, which may be attributed to measurement variations.

**Table 2:** Comparison of performance evaluation metrics for training and testing datasets in the ANN model.

ANN model	R^2^	MAE	RMSE	MSE	AARD (%)
Training set	0.991	0.0141	0.0220	0.0005	26.238
Testing set	0.986	0.0171	0.0273	0.0007	28.054

**Figure 5:**
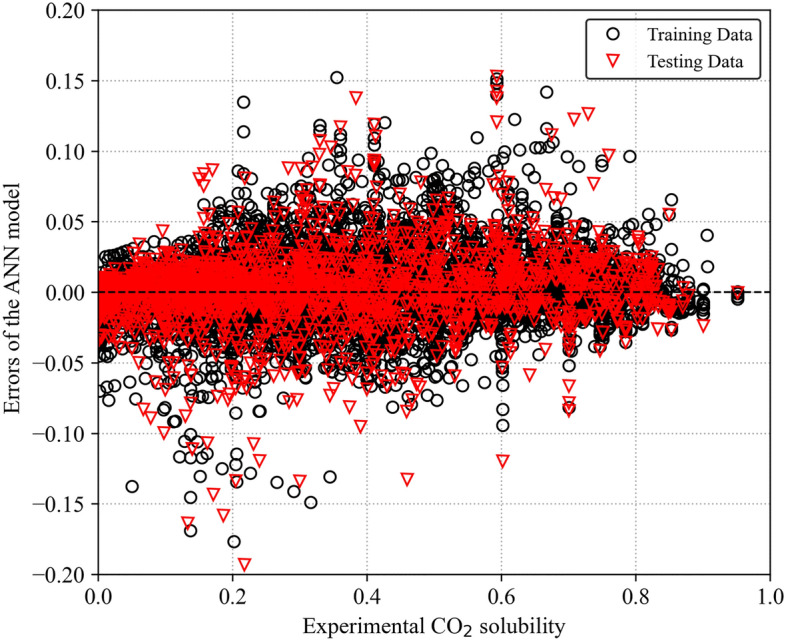
ANN model errors for predicting CO_2_ solubility.

**Figure 6:**
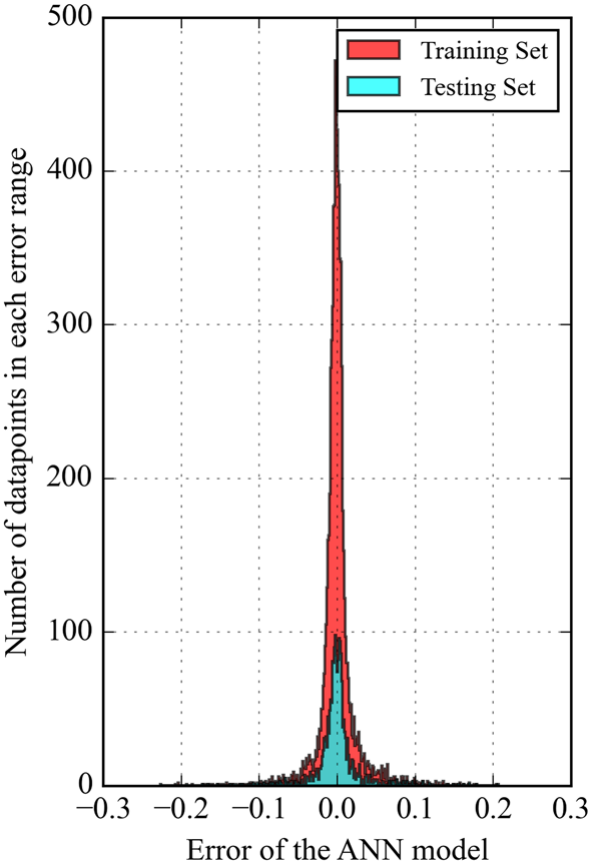
Distribution of prediction error of ANN model.

The discrepancy between predicted and experimentally measured values is analyzed to assess model performance. Figure 5 represents the distribution of errors between predicted and experimental solubilities. ${(x}_{c{o}_{2}}^{predicted}-{x}_{c{o}_{2}}^{experimental})$ for the ANN model. Most data points fall within a narrow range of − 0.05 to 0.05, indicating a smooth distribution close to zero. However, a few outliers exhibited higher error values. Figure 6 presents a histogram of the error distribution for the ANN model to provide further insights into the range of predicted errors. It is seen that the error distribution is mainly concentrated around zero, with minimal deviation. This suggests the ANN model accurately predicts CO_2_ solubility across various temperatures and pressures in ILs.

### LSTM model

This study aims to implement a Long Short-Term Memory (LSTM) model for predicting CO_2_ solubility in ILs. LSTM models have seen limited application in this field. Deng et al.^31^ have used a classic Recurrent Neural Network (RNN) model for predicting CO_2_ solubility in ILs, employing a dataset of 180 data points. While RNNs are typically used for time series problems to analyze long-term dependencies, their applicability to regression problems has been demonstrated^31^. This study significantly improves over previous work by Song et al.^30^ by replacing the SVM model with an LSTM neural network model. This substitution leads to a more accurate prediction of CO_2_ solubility in ILs.

**Figure 7:**
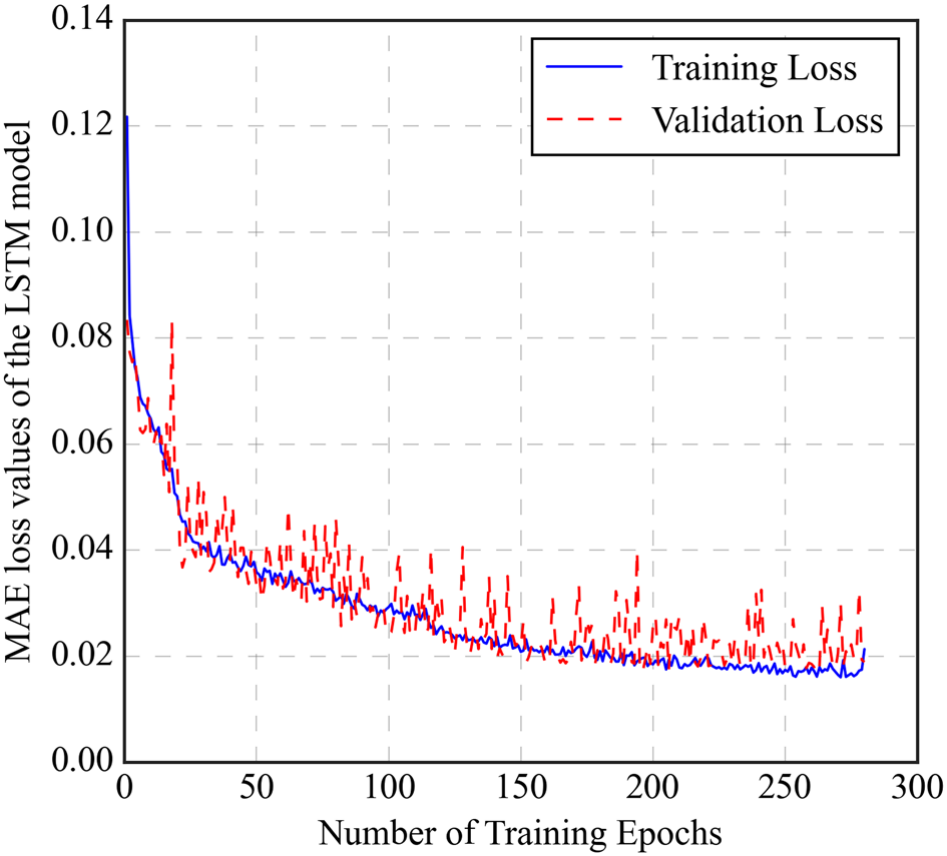
Mean absolute error (MAE) loss curves for the LSTM model showing training and validation performance over epochs.

**Figure 8:**
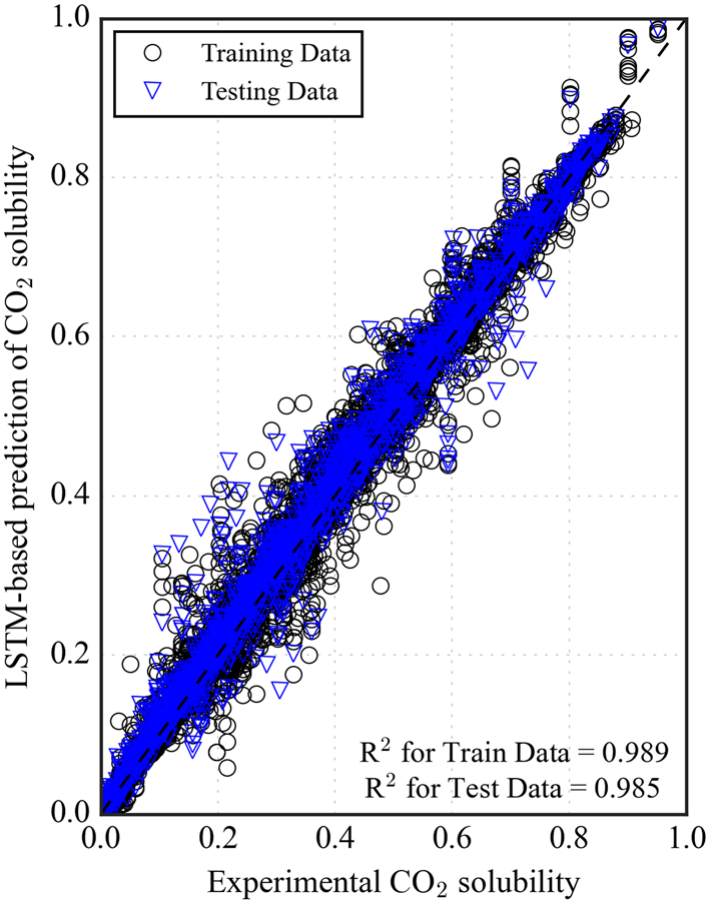
Comparison of experimental and LSTM predicted CO_2_ solubility.

The LSTM model was structured with a dual-layer configuration, each containing 64 neurons (Supplementary Fig. S2). The widely used "tanh" activation function is the default choice for all hidden layers^34^. Typically, dropout functions are utilized to address overfitting issues in model^54^. Even though dropout functions are implemented to prevent overfitting in models. The LSTM model showed no signs of overfitting and was performing adequately; dropout was not incorporated. The Adam optimizer with a learning rate of 0.001 was used to optimize the training of the LSTM model. For hyperparameter tuning, various batch sizes were tested to train the dataset, and a batch size of 16 and 280 epochs yielded the best results. The training and validation loss curves observe the determination of the number of epochs. Figure 7 demonstrates the MAE loss curves for the training and data validation. The training data reveals a significant drop in MAE errors (blue line), highlighting the robust learning capability of the LSTM model. The MAE loss for the validation data (red dashed line) shows the stability of the model by avoiding overfitting. Table 3 provides evaluation metrics to compare the efficiency of training and testing the LSTM model. The LSTM model achieved an R^2^ of 0.985 and an MAE of 0.0175 on the testing data, with differences from the training data by 0.41% and 11.9%, respectively. The predicted CO_2_ solubilities are compared with the experimental values. They are presented in Fig. 8. The data points for the training (black circle) and testing datasets (blue triangle) are evenly distributed around the diagonal line, indicating good agreement between the predicted and experimental CO_2_ solubility values.

**Table 3:** Comparison of performance evaluation metrics for training and testing datasets in the LSTM model.

LSTM model	R^2^	MAE	RMSE	MSE	AARD%
Training set	0.989	0.0155	0.0258	0.0007	8.154
Testing set	0.985	0.0174	0.0293	0.0009	10.793

**Figure 9:**
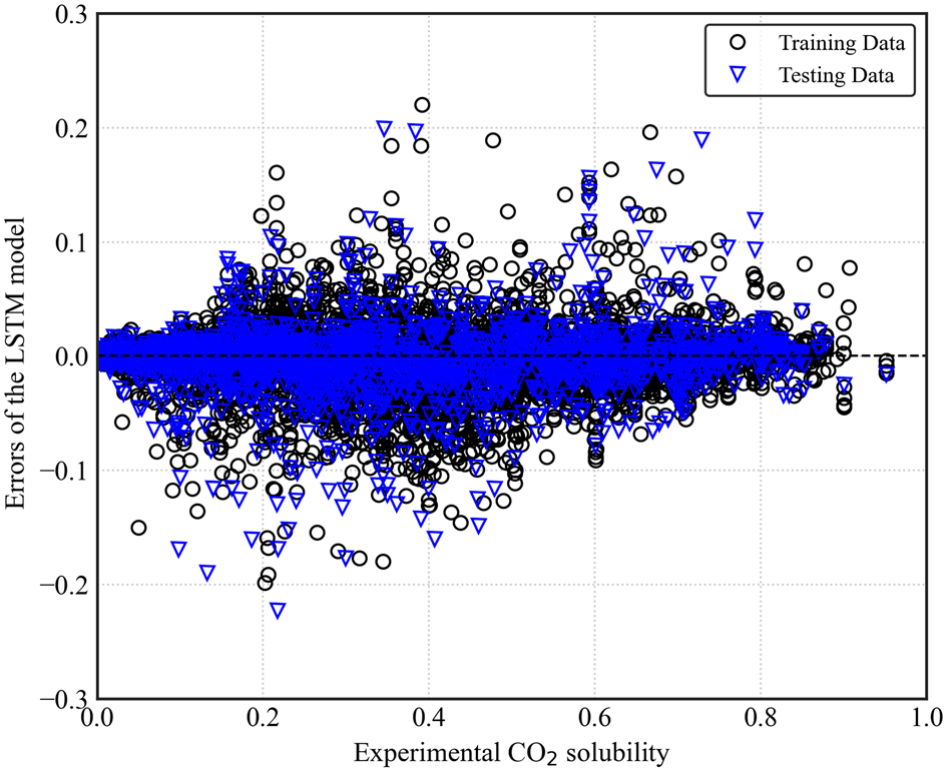
LSTM model errors for predicting CO_2_ solubility.

**Figure 10:**
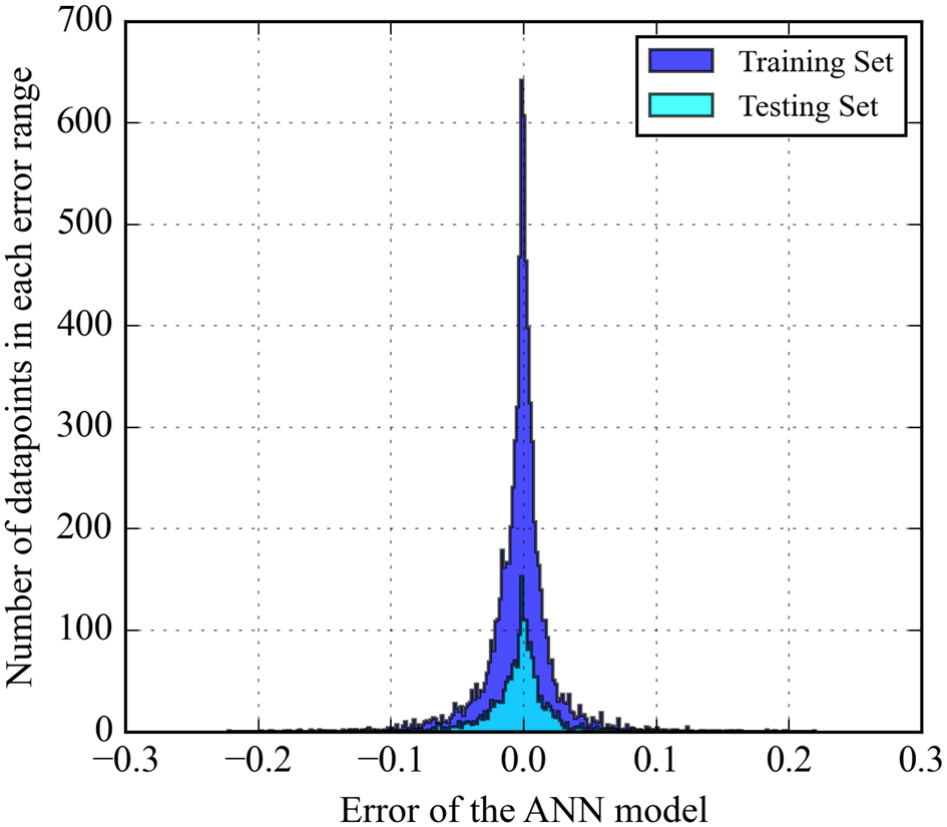
Distribution of prediction errors of the LSTM model.

Figure 9 depicts the distribution of errors between the predicted and experimental CO_2_ solubility values for the LSTM model on both the training and testing datasets. ${(x}_{c{o}_{2}}^{predicted}-{x}_{c{o}_{2}}^{experimental})$. The LSTM model also demonstrates the favorable error distribution for training and testing data, with errors falling from − 0.1 to 0.1 and exhibiting a consistent distribution centered around zero. This suggests good accuracy in predicting CO_2_ solubility; however, it is worth noting that the ANN model achieves a slightly lower error margin. Figure 10 utilizes histograms to provide a more granular visualization of the error distribution for the LSTM model. The histograms reveal minimal deviations from zero, indicating that the model predicts CO_2_ solubility accurately.

### Models comparison

A comprehensive evaluation compares the performance and computational efficiency of ANN and LSTM models for predicting CO_2_ solubility in ILs. This evaluation considers accuracy, training time (CPU usage), and memory expenditure during training. The computational cost of neural network models during training is analyzed by comparing their CPU time (seconds) and memory consumption (Mebibytes, MiB).

**Figure 11:**
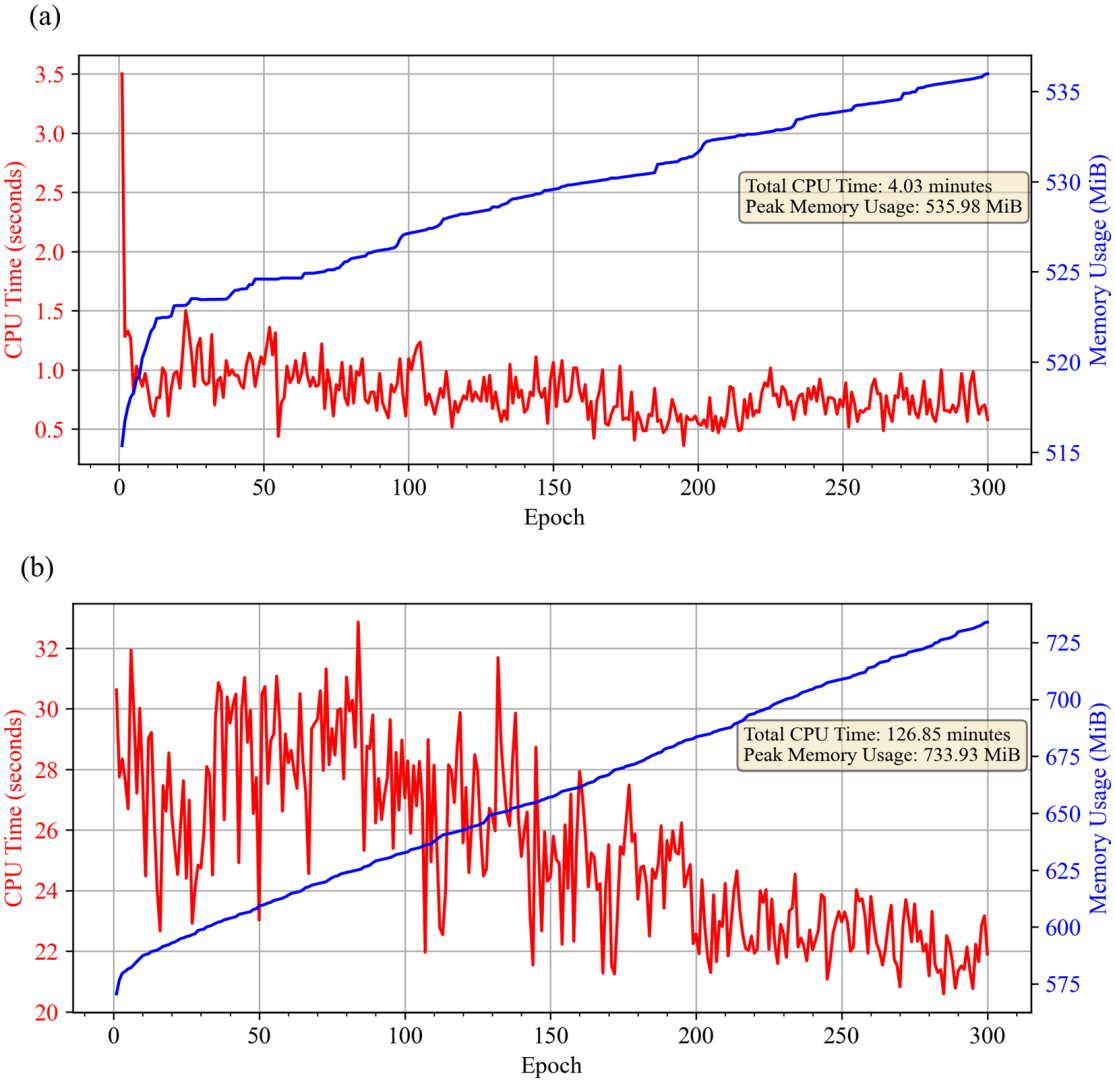
CPU time and memory usage during model training: (**a**) ANN model (**b**) LSTM model.

Figure 11 presents the graphical representation of CPU time and memory usage over the training epochs for the ANN and LSTM models. In terms of CPU time, the ANN model proves to be much more efficient. Each training epoch for the ANN model takes approximately 1 s (Fig. 11a), whereas the LSTM model requires a significantly longer time, averaging between 20 and 30 s per epoch (Fig. 11b). The total CPU time of the ANN model (4.03 min) is 31 times faster than that of the LSTM model (126.85 min) during the model's training. In comparing peak memory usage between ANN and LSTM models, the LSTM model consumed the most memory, reaching a peak of 733.93 MiBs at the end of the training, followed by the ANN model, which peaked at 535.98 MiBs. LSTMs incorporate memory cells that store past information, resulting in a larger memory footprint compared to the more straightforward layer-based structure of ANNs. LSTMs are inherently more complex architectures, including memory cells and gates (input, output, and forget) to control information flow and contribute to a higher computational load during training.

Table 4 summarizes the statistical comparison of ANN and LSTM models regarding model performance and error ranges. The ANN model performed slightly better than the LSTM model in terms of prediction accuracy. The R^2^ values of testing data in the ANN and LSTM models are 0.986 and 0.985, respectively. The MAE of the ANN model is 2.3% lower than the LSTM. Although both models have demonstrated excellent performance, the ANN model outperforms the LSTM model regarding computational cost and efficiency.

**Table 4:** Statistical comparison of ANN and LSTM models.

Models	R^2^	MAE	RMSE	MSE	AARD (%)
ANN	0.986	0.0172	0.0274	0.0007488	28.05
LSTM	0.985	0.0175	0.0294	0.0008616	10.80

Regarding the AARD values, it is worth noting that the LSTM model (10%) exhibits less deviation than the ANN model (28%). Initially, the ANN model recorded an AARD value of 57.5%, which was later reduced to 28.05% by increasing the number of hidden layers from 1 to 3 and adjusting the neuron count. The higher AARD percentage can be attributed to using a large dataset with diverse input parameters.

**Figure 12:**
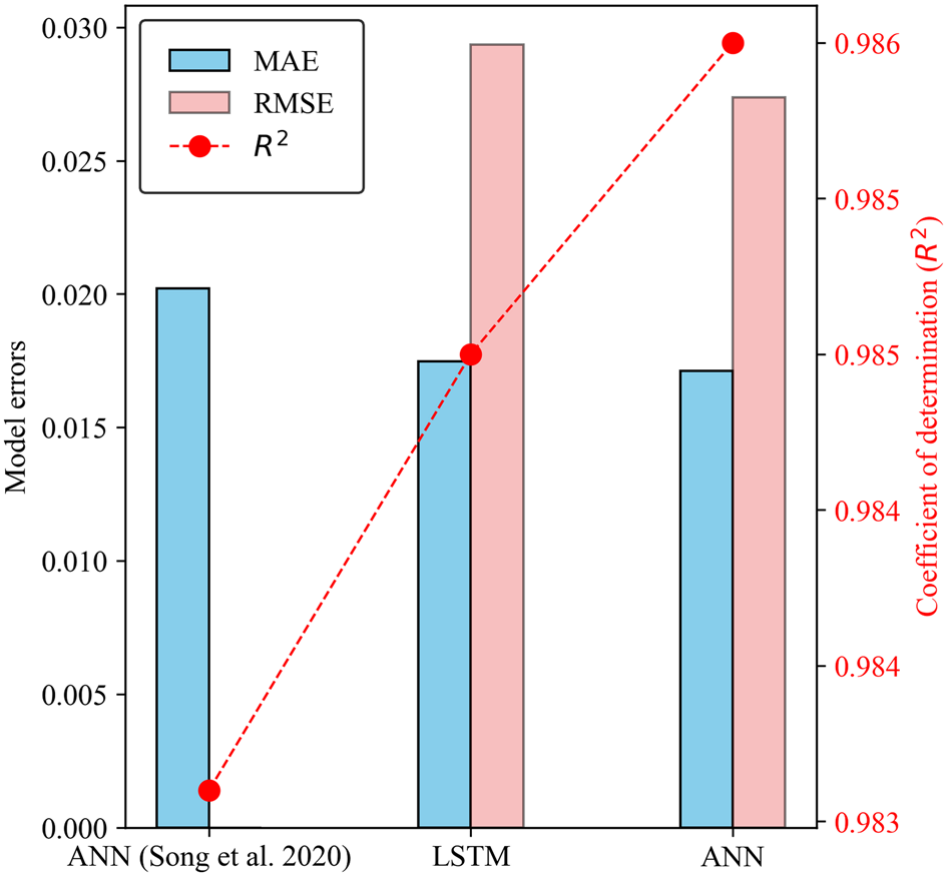
Performance comparison of the ANN and LSTM models with the ANN-GC model developed by Song et al.^30^.

**Figure 13:**
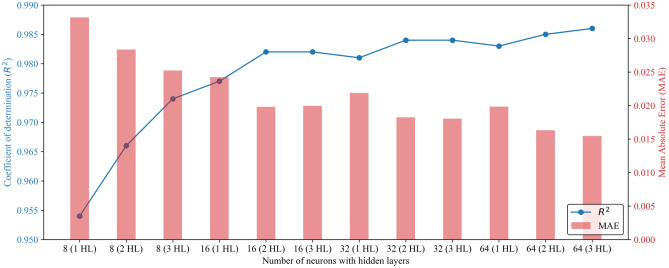
ANN model accuracy with different numbers of neurons and hidden layers.

Song et al.^30^ developed an ANN-GC model using the current dataset to predict the CO_2_ solubility in ILs. Figure 12 compares evaluation metrics between the current ANN and LSTM models and the ANN-GC model from the previous study^30^. The LSTM and ANN models slightly outperformed the ANN-GC model in terms of prediction accuracy. Specifically, the prediction accuracy of the current ANN model increased by 0.2%, accompanied by a 13% reduction in MAE compared to the ANN-GC model^30^. Table 5 compares the methodology used for ANN modelling in this study with the previous study^30^. This study adopts the ReLU activation function due to its computational efficiency and effectiveness with large datasets. It performs better in capturing non-linear patterns and gradients, making it well-suited for a wide range of problems. It is worth noticing that Song et al.^30^ achieved a significantly higher accuracy with 7 neurons in the hidden layer compared to this study, which utilized 64 neurons in each hidden layer. The optimal number of neurons is the most crucial step in designing neural networks, especially for a large dataset. Using a higher number of neurons and more hidden layers is generally preferred for larger datasets. This approach allows the neural network to learn and model big data's complex patterns and relationships more effectively. This study lacks information about the hyperparameter tuning and optimization processes. This study investigated the training and testing accuracy by adjusting the learning rate (using the Adam optimizer) and the batch size for model training. Figure 13 visualizes the effect of varying the number of neurons and hidden layers on ANN model accuracy. Optimal results were achieved by configuring 3 hidden layers and 64 neurons in each layer. Optimization aims to minimize the discrepancies between the predicted and actual outputs. As observed in Fig. 13, adjusting the number of hidden layers and neurons significantly reduced prediction errors.

**Table 5:** Statistical comparison of ANN-GC model^30^ with this study for the ANN model.

	Song et al*.* ^30^	This study
Activation function	Transig and Purelin	ReLU
Hidden layers	1	3
Neurons per hidden layer	7	64
R^2^	0.984	0.986
MAE	0.0202	0.0172

A study by Deng et al.^31^ employed an ANN model and achieved a high R^2^ of 0.999. However, their model was trained on a relatively small dataset of 218 data points for only 13 types of ILs. This limited data size might contribute to high accuracy, as smaller datasets can sometimes lead to overfitting. Additionally, their ANN architecture utilized a 7-layer network with many neurons ranging from 500 to 1, decreasing to 1 in the final layer. While this complex architecture may have performed well on their specific dataset, their study did not explicitly evaluate the impact of the specific number of neurons on model performance.

In addition to the DL models, traditional ML regression techniques, namely Random Forest (RFR) and Gradient Boosting Regression (GBR), were employed on this comprehensive dataset. Both RFR and GBR achieved R^2^ values of 0.974 and 0.966, respectively. A detailed visualization of the predicted values and their associated error ranges for both models is presented in Supplementary Fig. S3 of the supplementary materials. A review of multiple literature sources was conducted to achieve a comprehensive overview of the prediction accuracy of models concerning statistical parameters, the number of data points, and the variety of ILs utilized for predicting CO_2_ solubility. Table 6 compares the performance of various machine learning and thermodynamics-based models for CO_2_ solubility prediction in ILs. Interestingly, it is observed that models with higher R^2^ values and lower AARD values are often associated with smaller data points and a lower number of ILs in their respective studies. Despite the challenges associated with larger datasets, studies conducted by Venkatraman and Alsberg^28^ demonstrate promising results with a higher number of ILs and data points. Their RF and CTREE models achieved R^2^ values of 0.92 and 0.82, respectively. Song et al.^30^ reported the most extensive dataset for ILs. In their work, authors developed ANN-GC and SVM-GC models, yielding reliable R^2^ values of 0.9836 and 0.9783, respectively. Among the literature studies surveyed, Mesbah et al.^55^ introduced the MLP-ANN model, which achieved the highest R^2^ value of 0.9987 and the lowest AARD value of 1.8416. This model was evaluated using a dataset comprising 20 ionic liquids (ILs) and 1386 data points.

**Table 6:** Various model comparisons for CO_2_ solubility in ILs.

Model name	Data points	No. of ILs	R^2^	MAE	MSE	RMSE	%AARD	References
MLPNN	548	1	0.98631	–	0.00094	0.03066	7.21	^55^
CFNN	548	1	0.98808	–	0.00080	0.02829	6.88	
GRNN	548	1	0.99079	–	0.00062	0.02490	13.6	
RBF	548	1	0.98516	–	0.00100	0.03159	13.5	
ANFIS	548	1	0.98732	–	0.00085	0.02919	7.95	
LS-SVM	548	1	0.98428	–	0.00106	0.03253	9.71	^40^
DT	1668	40	0.94	–	–	–	21.24	^56^
RF	1668	40	0.96	–	–	–	12.05	
LSSVM	1668	40	0.75	–	–	–	31.01	
MLR	1668	40	0.55	–	–	–	40.48	
COSMO-RS	10,848	185	0.71	0.12	–	0.19	–	^28^
RF	10,848	185	0.92	0.04	–	0.07	–	
CTREE	10,848	185	0.82	0.10	–	0.07	–	
DNN	218	13	0.984	0.291	–	0.757	–	^31^
CNN	218	13	0.999	0.145	–	0.206	–	
RNN	218	13	0.988	0.25	–	0.651	–	
XG Boost	218	13	0.981	0.175	–	0.586	–	
MLP-ANN	1386	20	0.9987	–	0.6293	–	1.8416	^55^
ANFIS	728	14	0.9972	–	0.00294	0.05423	–	^37^
MLP-ANN	728	14	0.6989	–	0.00013	1.00145	–	
PR-EOS	728	14	0.9376	–	0.00270	0.05198	–	
SRK-EOS	728	14	0.9336	–	0.00558	0.07468	–	
MLR	32	32	0.892	–	–	0.372	6.33 (ARD)	^57^
LS-SVM	32	32	0.962	–	–	0.221	4.42 (ARD)	
PSO-ANFIS	1119	11	0.9397	–	0.3910	–	14.1286	^58^
CSA-LSSVM	1119	11	0.9846	–	0.0999	–	3.0410	
BP	544	9	0.9982	0.0068	–	0.0090	–	^59^
SVM	544	9	0.9933	0.0105	–	0.0174	–	
ELM	544	9	0.9961	0.0093		0.0136		
Linear fusion model I	544	9	0.9983	0.0062	–	0.0090	–	
Linear fusion model II	544	9	0.9985	0.0060	–	0.0084	–	
GMDH	4726	60	0.9043	–	–	0.0765	–	^60^
ANN-GC	10,116	124	0.9836	0.0202	–	–	–	^30^
SVM-GC	10,116	124	0.9783	0.0240	–	–	–	
ANN	10,116	124	0.986	0.0172	0.00074	0.02736	28.054	This study
LSTM	10,116	124	0.985	0.0175	0.00086	0.02935	10.793	
RF	10,116	124	0.974	0.0232	0.00138	0.03719	11.875	
GBR	10,116	124	0.966	0.0305	0.00182	0.04277	67.82	

### Global sensitivity analysis (GSA)

CO_2_ solubility in ILs is strongly influenced by input parameters such as temperature, pressure, and the presence of functional groups. Blanchard et al.^61^ demonstrated efficient CO_2_ dissolution in ILs at 25 °C and pressures up to 40 MPa. Extensive research has explored CO_2_ absorption with ILs, encompassing both conventional ILs relying on physisorption and functionalized ILs utilizing chemisorption mechanisms^14^. Generally, for conventional ILs, the anions are more effective for CO_2_ absorption, while cations have relatively low effects.

The solubility of CO_2_ in ILs has been investigated through Global Sensitivity Analysis (GSA) to assess the relative impacts of process parameters, including temperature, pressure, and various functional groups. This analysis aims to ascertain the significance of these factors on the solubility behaviour of CO_2_ in ILs. GSA is a robust approach that evaluates the influence of input parameters on outputs by allowing all inputs to fluctuate within predefined ranges^47^, providing valuable insights into the consequences of input variations on the overall system behaviour.

For GSA, two widely used techniques, Sobol sensitivity analysis^46^ and Morris sensitivity analysis^48^, were applied to analyze the effect of input variables on CO_2_ solubility in ILs. In the Sobol method, the total sensitivity index (*S*_*T*_) is utilized to assess the overall impact of an input variable on CO_2_ solubility. The *S*_*T*_ quantifies an input variable's total effect on the model output. On the other hand, the Morris method employs the μ index, which represents the average effect of each input variable over the sampled parameter space. It quantifies the average change in the model output when a variable is perturbed while holding other variables constant. Higher μ values indicate a more significant influence of the variable on the model output.

**Figure 14:**
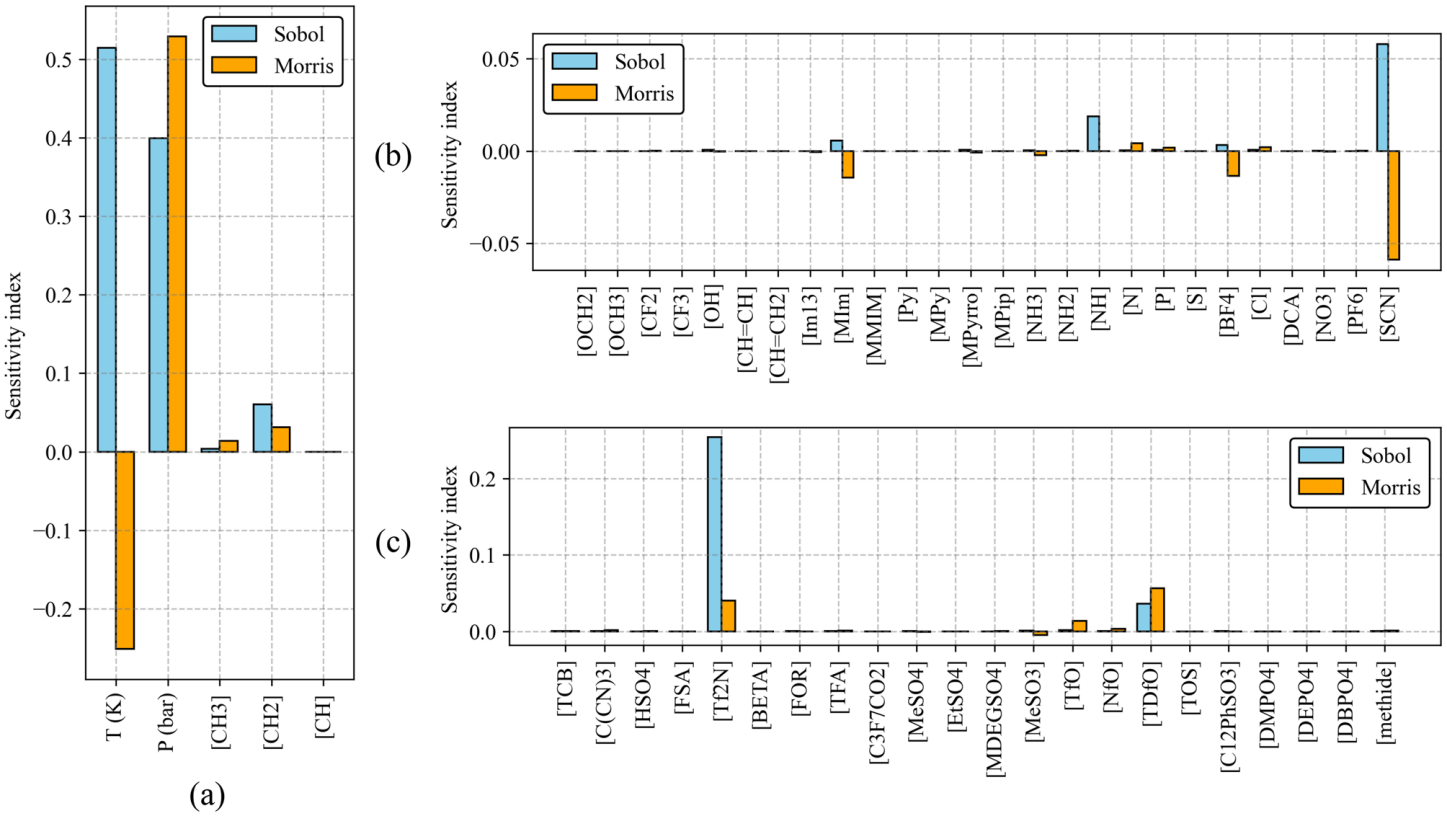
Sobol and Morris sensitivity indices of temperature and pressure with 53 functional groups.

Figure 14 presents the results of both Sobol and Morris global sensitivity analysis for temperature (*T*), pressure (*P*), and the functional groups. While both methods provide valuable insights, they may present slightly different perspectives. Pressure emerges as a dominant factor affecting CO_2_ solubility. This is evident in Fig. 14a, where both methods indicate a significant sensitivity index for pressure. This means changes in pressure have a strong impact on predicted CO_2_ solubility values. The temperature (*T*) index is positive for the Sobol analysis and negative for the Morris analysis. The Sobol sensitivity analysis unexpectedly assigns a positive value to the *T* index. This finding seemingly contradicts the established knowledge that temperature has a negative impact on CO_2_ solubility (i.e., higher temperature leads to lower CO_2_ solubility). The Sobol method is sensitive to non-linear relationships between input parameters and the output. The true relationship between temperature and CO2 solubility may be non-linear within the range of your data. The positive Sobol index might capture an initial increase in CO_2_ solubility followed by a decrease at higher temperatures, which a simple negative index would not reflect. Jerng et al. have indicated that the CO_2_ solubility decreases with increasing temperature^62^. The Morris method suggests a negative correlation between temperature and CO_2_ solubility, aligning with the observation that CO_2_ solubility increases as temperature decreases. Figure 14b,c display the sensitivity indices for various functional groups. The graphs indicate that some functional groups have a minimal influence on CO_2_ solubility, whereas others demonstrate a negative impact. Supplementary Table S1 (Supplementary Material) presents the sensitivity index values for each parameter across the dataset, as determined by the Sobol and Morris sensitivity analysis methods.

When dealing with extensive datasets that include numerous input variables, the Morris method could be a preferable initial option over the Sobol sensitivity analysis. Due to its faster execution and lower computational demand, the Morris method is particularly beneficial for large-scale data processing, enabling rapid analysis with modest resource consumption. The Morris method serves as a valuable tool for initial screening. It can efficiently identify the most influential parameters (such as pressure and temperature) while filtering out those with a lower impact (certain functional groups).

This study offers a valuable combination of high accuracy, efficiency, and insights into model interpretability using deep learning models. Still, these models' ability to generalize to other solutes or liquid types remains unverified. Another limitation to consider is the computational cost of the LSTM model. Although both models achieve high accuracy, the LSTM model requires significantly more training time and memory resources than the ANN model. This could limit its applicability in real-world scenarios where computational power or hardware resources might be restricted.

## Conclusions

This study investigated the potential of deep learning models for predicting CO_2_ solubility in ionic liquids (ILs). A comprehensive dataset containing over 10,116 CO_2_ solubility measurements covering 164 different ILs under varying temperatures and pressures was used to train two deep neural network models: an Artificial Neural Network (ANN) and a Long Short-Term Memory (LSTM) network. The hyperparameter tuning, optimization, and validation strategy were conducted to evaluate the model performance comprehensively. The efficiency of the ANN and LSTM models was compared by analyzing their computational demands and memory consumption throughout the training process. Both models demonstrated remarkable accuracy in predicting CO_2_ solubility. The ANN model achieved a high R^2^ of 0.985 in just 4 min of training, consuming 535 MiB of memory. The LSTM model required significantly more training time (approximately 126 min) and consumed more memory (735 MiB) to achieve a comparable R^2^ of 0.984. This difference can be attributed to the LSTM architecture's inherent complexity in handling sequential data. The ANN model achieved a 13% lower error rate than a previous study that used an ANN-GC model on a similar dataset. In this study, the size of neurons is optimized within the ANN model to achieve this higher accuracy and lower error rate. A review of existing literature on the prediction models developed for CO_2_ capture in ILs was conducted to gain insights into the relationship between model performance and characteristics of ILs.

Sobol and Morris, sensitivity analysis methods, were employed to investigate the relative importance of input parameters on CO_2_ solubility in ILs. The Morris sensitivity analysis identified pressure and temperature as having the most significant influence on CO_2_ solubility in ILs, aligning well with experimental observations. The Morris method is a computationally efficient and easy-to-interpret technique for initial sensitivity analysis, particularly suitable for large datasets. The sensitivity analysis results provided valuable insights into the model's sensitivity to different parameters and helped identify the key factors driving the CO_2_ solubility.

This study offers significant advancements in predicting CO_2_ solubility in ILs using deep learning models. The high accuracy and efficiency of the ANN model make it a promising tool for streamlining the screening process of ILs for CO_2_ capture applications. This paves the way for further exploration of deep learning approaches for similar prediction tasks in CO_2_ capture research and potentially extends its application to other areas of material science.

### Supplementary Information

Supplementary Information.

## Data Availability

The datasets used and analyzed during the current study are available from the corresponding author upon reasonable request.

## List of symbols

*a*: Activations (or the total inputs) for the neurons in the layers
*b*: Bias vectors for the layers
${\widetilde{C}}_{t}$: A memory state (a vector in an LSTM cell)
*C*_*t*_: Cell state in an LSTM cell
*D*: Total variance in sensitivity analysis
*D*_*i*_: First-order variance contribution of parameter *i*
*D*_*ij*_: Second-order variance contribution from the interaction between parameters *i* and* j*
*D*_*ijk*_: Third-order variance contribution from the interaction between parameters *i, j,* and* k*
*EE*_*i*_: Elementary effect for the parameter *i*
*f*_*1*_: Activation functions for the first layer
*f*_*2*_: Activation functions for the second layer
*f*_*t*_: Forget the gate in an LSTM cell
*h*_*i*_: Hidden state of the LSTM cell
*i*_*t*_: Input gate in an LSTM cell
*o*_*t*_: Output gate in an LSTM cell
*S*_*i*_: First-order sensitivity index for parameter *i*
*S*_*Ti*_: Total-order sensitivity index for parameter *i*
*P*: Pressure (Pa)
*p*: Input vector to the neural network
*R*: Coefficient of determination [–]
*T*: Temperature (K)
*W*: Weight matrices
*tanh*: Hyperbolic tangent activation function
*σ*: Sigmoid activation function
*μ*: Average of elementary effects for a parameter in Morris sensitivity analysis

## Abbreviations

ANN: Artificial neural network
ANFIS: Adaptive neuro-fuzzy inference system
AARD: Average absolute relative deviation (AARD)
BP: Back Propagation (commonly used in neural networks)
CO_2_: Carbon dioxide
CNN: Convolutional neural network
CSTREE: Conditional inference trees
COSMO-RS: Conductor-like screening model for real solvents
CAMD: Computer-aided molecular design
CFNN: Cascade forward neural network
CSA-LSSVM: Cuckoo search algorithm least squares support vector machine
CPU: Central processing unit
DT: Decision tree
DNN: Deep neural network
DEA: Diethanolamine
ELM: Extreme learning machine
GC: Group contribution
GMDH: Group method of data handling
GWO: Grey Wolf Optimization
GRNN: General regression neural network
GBR: Gradient boost regressor
H_2_S: Hydrogen sulfide
IL: Ionic liquids
IFC: Ionic fragments contribution
LS-SVM: Least squares support vector machine
MAE: Mean absolute error
MSE: Mean squared error
MiB: Mebibytes
MLPNN: Multilayer perceptron neural network
MLP-ANN: Multilayer perceptron artificial neural network
MLR: Multiple linear regression
MEA: Monoethanolamine
MDEA: Methyldiethanolamine
PSA: Pressure swing absorption
PSRK: Peng–Robinson–Stryjek–Vera
PR-EOS: Peng–Robinson equation of state
PLSR: Partial-least-squares regression
PSO: Particle swarm optimization
QSPR: Quantitative structure–property relationship
ReLU: Rectified linear activation function
RNN: Recurrent neural network
RBF: Radial basis function (network or kernel)
RF: Random forest
RMSE: Root mean square error
SRK-EOS: Soave–Redlich–Kwong equation of state
SVM: Support vector machine
SAFT: Statistical associating fluid theory
SSA: Sparrow search algorithm
TSA: Temperature swing adsorption
XG Boost: EXtreme gradient boosting
[BF_4_]: Tetrafluoroborate
[DCA]: Dicyanamide
[PF_6_]: Hexafluorophosphate
[Cl]: Chloride
[NO_3_]: Nitrate
[C(CN)_3_]: Tricyanomethanide
[Tf_2_N]: Bis(trifluoromethylsulflonyl)amide
[HSO_4_]: Hydrogen sulfate
[MeSO_4_]: Methylsulfate
