# Supplementary material for: Prediction of CO2 solubility in Ionic liquids for CO2 capture using deep learning models
Source: Sci Rep. 2024 Jun 26;14:14730. doi: 10.1038/s41598-024-65499-y (PMC11208552; doi:10.1038/s41598-024-65499-y)
Supplement: Supplementary file 1 — Supplementary Information. [file 41598_2024_65499_MOESM1_ESM.docx]

**Prediction of CO_2_ solubility in Ionic liquids for CO_2_ capture using deep learning models**

Mazhar Ali^1^, Tooba Sarwar^1^, Nabisab Mujawar Mubarak^2,3*^, Rama Rao Karri^2,4*^, Lubna Ghalib^5^, Aisha Bibi^6^, Shaukat Ali Mazari^1^*

^1^Department of Chemical Engineering, Dawood University of Engineering & Technology, Karachi, Pakistan

^2^Petroleum and Chemical Engineering, Faculty of Engineering, Universiti Teknologi Brunei,

Bandar Seri Begawan, BE1410, Brunei Darussalam

^3^Department of Chemistry, School of Chemical Engineering and Physical Sciences, Lovely Professional University, Phagwara-144411, Punjab (India)

^4^INTI International University, 71800 Nilai, Negeri Sembilan, Malaysia

^5^Materials Engineering Department, Mustansiriayah University, 14022 Baghdad, Iraq

^6^Department of Education, NUML, Islamabad, Pakistan

* Corresponding authors: mubarak.mujawar@utb.edu.bn; kramarao.iitd@gmail.com; shaukat.mazari@duet.edu.pk

**S1. Graphical representation of ANN model architecture**


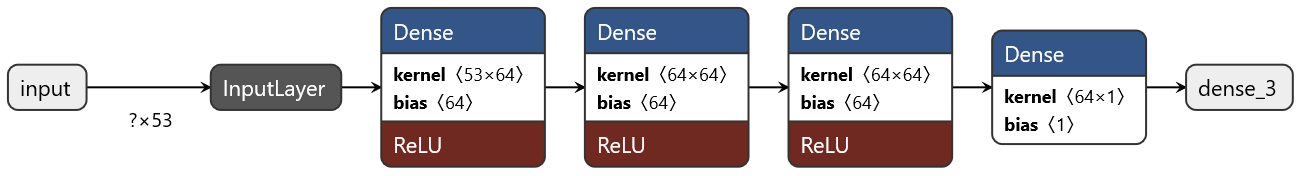


**Fig S1:** Visualization of the architecture of ANN model

**S2. Graphical representation of LSTM model architecture**


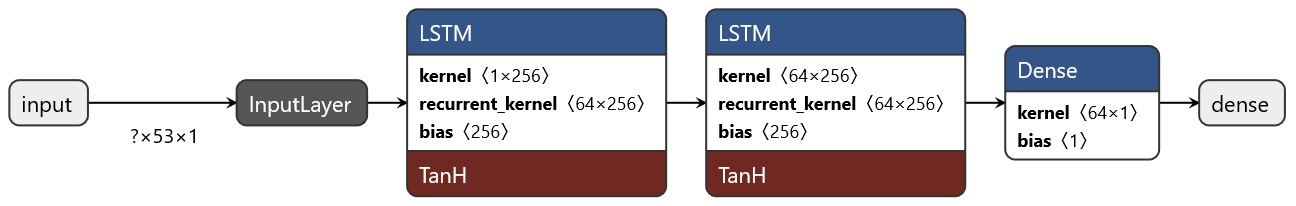


**Fig S2:** Visualization of the architecture of LSTM model

**S3. Random Forest Regressor (RFR) and Gradient Boost Regressor (GBR) model performance for the 10,116 CO_2_ solubility data**

**Fig. S3:** ML models performance (a) experimental vs predicted values (b) model errors.

**S4. Sobol and Morris sensitivity indices for input parameters in ILs**

**Table S1:** Sobol and Morris sensitivity indices for input parameters

| Variables | $\boldsymbol{\mu}$ (Morris) | ST (Sobol) | Variables | $\boldsymbol{\mu}$ (Morris) | ST (Sobol) |
| --- | --- | --- | --- | --- | --- |
| T (K) | -0.25763 | 0.4890982 | [NH_3_] | -0.001884 | 0.000187244 |
| P (bar) | 0.472864 | 0.3828027 | [NH_2_] | 0.000275 | 5.28E-06 |
| [CH_3_] | 0.016057 | 0.003523184 | [NH] | 0 | 0.02210994 |
| [CH_2_] | 0.024785 | 0.07402076 | [N] | 0.004385 | 0.000244433 |
| [CH] | 0 | 9.07E-12 | [P] | 0.002005 | 0.000475535 |
| [OCH_2_] | 0.000123 | 2.41E-05 | [S] | 0 | 0 |
| [OCH_3_] | 0 | 1.07E-07 | [BF_4_] | -0.00418 | 0.000769815 |
| [CF_2_] | 2.30E-05 | 5.61E-06 | [Cl] | 0.001028 | 0.000502043 |
| [CF_3_] | 2.40E-05 | 7.40E-06 | [DCA] | -4.40E-05 | 2.03E-06 |
| [OH] | -0.000321 | 0.000605583 | [NO_3_] | -0.000154 | 2.21E-05 |
| [CH=C] | 0 | 2.94E-07 | [PF_6_] | 0.000119 | 5.43E-06 |
| [CH=C] | NaN | NaN | [SCN] | -0.073202 | 0.08793944 |
| [Im13] | -0.000285 | 8.07E-06 | [TCB] | 0.000409 | 4.87E-05 |
| [MIm] | -0.016326 | 0.00518625 | [C(CN] | NaN | NaN |
| [MMIM] | 0 | 2.66E-06 | [HSO_4_] | 4.00E-06 | 1.09E-07 |
| [Py] | 2.00E-06 | 2.05E-06 | [FSA] | 0 | 1.11E-09 |
| [MPy] | -3.00E-06 | 3.04E-09 | [Tf_2_N] | 0.039044 | 0.2778954 |
| [MPyr] | -0.001374 | 0.00093025 | [BETA] | 0 | 1.01E-10 |
| [MPip] | 0 | 2.60E-11 | [FOR] | -0.000191 | 0.000661971 |
| [C_3_F_7_] | NaN | NaN | [TDfO] | 0.048748 | 0.03754035 |
| [MeSO] | NaN | NaN | [TOS] | -0.000206 | 5.24E-06 |
| [EtSO] | NaN | NaN | [C12P] | NaN | NaN |
| [MDEG] | NaN | NaN | [DMPO] | NaN | NaN |
| [MeSO | NaN | NaN | [DEPO] | NaN | NaN |
| [TfO] | 0.016968 | 0.001890051 | [DBPO] | NaN | NaN |
| [NfO] | 0.003867 | 0.000437077 | [meth] | 0.00053 | 4.48E-05 |
| [TFA] | 0.000271 |  |  |  |  |
